# AlScN Pseudosubstrates for High Indium Content InGaN Alloy Epitaxy

**DOI:** 10.1021/acsami.5c14209

**Published:** 2025-10-23

**Authors:** Jörg Schörmann, Mario F. Zscherp, Silas A. Jentsch, Martin Becker, Markus Stein, Florian Meierhofer, Christoph Margenfeld, Fabian Winkler, Andreas Beyer, Kerstin Volz, Andreas Waag, Sangam Chatterjee

**Affiliations:** † Institute of Experimental Physics I and Center for Materials Research, 9175 Justus-Liebig-University Giessen, Giessen D-35392, Germany; ‡ Nitride Technology Center, Institute of Semiconductor Technology, 26527 Technische Universität Braunschweig, Braunschweig D-38106, Germany; § Materials Science Center and Faculty of Physics, 9377 Philipps-University Marburg, Marburg D-35032, Germany

**Keywords:** AlScN, InGaN, molecular beam epitaxy (MBE), lattice-matched templates, red light emitting diodes

## Abstract

Nitride-based semiconductors are vital for efficient optoelectronic devices in the ultraviolet to green spectral range. However, producing red-emitting InGaN micro-LEDs is challenging due to lattice mismatch with traditional GaN substrates. This mismatch causes strain relaxation, compositional gradients, and defects in high-indium-content InGaN films. These issues severely limit device efficiency, and the potential of alternative substrates to address these challenges is not fully explored. Here, we show that Al_1–*x*_Sc_*x*_N pseudosubstrates with adjustable lattice parameters greatly improve lattice matching of InGaN. Using plasma-assisted molecular beam epitaxy, we grow 120 nm-thick, phase-pure Al_1–*x*_Sc_*x*_N layers (0.1 < *x*_Sc_ < 0.2). This enables high-quality deposition of In_0.28_Ga_0.72_N layers and a uniform indium distribution compared to growth directly on GaN. AlScN-supported films exhibit no compositional pulling effect common for conventional substrates. This uniformity is confirmed by room-temperature photoluminescence, showing a narrow emission at 538 nm. Our results demonstrate that AlScN pseudosubstrates are promising for future integrated red micro-LED devices.

## Graphical abstract

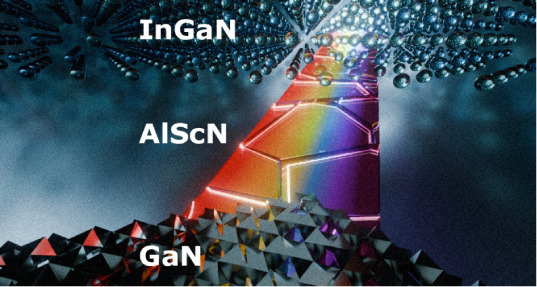

## Introduction

The outstanding material properties of (Al,Sc)N alloys, above all its high piezoelectric coefficient, are currently kindling intense research interest.^1,2^ Much discussed applications include surface acoustic wave (SAW) devices,^3,4^ as well as heterostructure field-effect transistors.^5–7^ More recently, AlScN films have been reported to be a ferroelectric III-nitride material.^8–10^ Consequently, AlScN also shows high potential in nonlinear optics.^11^ The advantages of this materials, however, extend beyond exploiting its ferroelectric properties.

Various growth techniques yield AlScN layers of high crystal quality including sputtering,^1,2,12,13^ metal–organic vapor phase epitaxy (MOVPE),^7,14^ and plasma-assisted molecular beam epitaxy (PAMBE).^5,15–18^ As the materials development is still in an early stage, large variation in the *a* and *c* lattice parameters of Al_1–*x*_Sc_*x*_N with increasing Sc composition are reported from the various growth techniques and means of quantifying the alloy composition.^1,2,5,12,13,17,19^ In particular, the large growth windows for Sc-containing compound semiconductors and their alloys render significant structural-design advantages compatible with the established nitrides device platform. For example, lattice matching to GaN for *x*_Sc_ ≈ 0.11 makes Al_1–*x*_Sc_*x*_N a prime candidate for integration into distributed Bragg reflectors.^20^ In this study, we propose the integration of AlScN with higher Sc content as a virtual substrate for lattice-matched subsequent high indium content InGaN thin films.

InGaN is a promising candidate material for improving the efficiency of red micro-LEDs. Typically, efficient large-area red LEDs are made from III-phosphides. However, III-phosphide LEDs are less efficient than those with III-nitrides when scaled down to a few μm and smaller due to longer carrier diffusion lengths and more severe surface recombination.^21,22^ In addition, heterogeneously integrating different materials such as III-nitrides and III-phosphides at the microscale adds another challenge to mass production due to complexities in transfer technology and difficulties in circuit design.^23^ In this regard, having high-efficiency blue, green, and red micro-LEDs using InGaN as the sole active material integrated on a single platform is highly desirable.

Red InGaN micro-LEDs (25 μm diameter) have been reported to achieve a practical external quantum efficiency of 5%.^24^ One of the major challenges in realizing efficient red InGaN LEDs is the large lattice mismatch between InGaN and GaN. It imposes significant limitations on the architecture of nitride devices, especially for red-emitting InGaN micro-LEDs.^25,26^ The fabrication of high-quality InGaN with high In content is particularly difficult due to the drastic differences between InN and GaN in bond strength and, hence, very different optimized growth temperatures: incorporating more In requires a low growth temperature and increases the lattice mismatch between InN and GaN which often leads to unwanted strain or even defect formation.^27^ This substantially limits the design freedom in nitride technology. AlScN is envisioned as a virtual substrate for the growth of lattice-matched InGaN to overcome these limitations by tuning the in-plane lattice parameter of AlScN.^28^ Growing high-quality AlScN on GaN with large mismatch is more straightforward than growing comparable InGaN, as the bond strengths of Sc–N and Al–N are substantially higher than In–N. Thus, AlScN has a large growth temperature window that is compatible with GaN or even InGaN epitaxy at low temperatures.^17,29^

In this work we show that Al_1–*x*_Sc_*x*_N can be used as a pseudosubstrate for high In-content In_*x*_Ga_1–*x*_N epitaxy. We achieved phase pure Al_1–*x*_Sc_*x*_N layers with Sc content of 20% and a thickness of 120 nm by plasma-assisted molecular beam epitaxy. The structural parameters, e.g., the in-plane lattice parameter and surface morphology of Al_1–*x*_Sc_*x*_N are determined and show comparable values to literature. To reveal the potential of AlScN as a pseudosubstrate, 100 nm thick In_*x*_Ga_1–*x*_N with *x*_In_ = 0.28 is grown both, on an Al_1–*x*_Sc_*x*_N-on-GaN layer as well as directly on a GaN template. Systematic examination of the structural properties demonstrates an extremely homogeneous film featuring a lower density of threading dislocations (TDs) when using the Al_1–*x*_Sc_*x*_N pseudosubstrate. Compositional pulling is only observed for In_*x*_Ga_1–*x*_N grown directly on GaN in XRD. These observations are corroborated by photoluminescence spectroscopy (PL) indicating single peak emission for InGaN/AlScN layers.

## Experimental Section

The epitaxial layers are grown in a Riber Compact12 MBE chamber. Gallium (7N), Al (6N), Sc (5N), and In (6N) are supplied from standard effusion cells. An Oxford Applied Research HD25 radio frequency plasma source supplies nitrogen at a flow rate of 0.7 sccm and a power of 200 W. Reflection high-energy electron diffraction (RHEED) provides in situ growth monitoring, while the sample growth temperature was measured by a pyrometer. We use commercial GaN-on-sapphire (10 × 10 mm^2^) substrates grown by metal–organic vapor phase epitaxy using a standard low-temperature GaN nucleation layer and subsequent high-temperature buffer. A 300 nm titanium backside coating was deposited using electron beam evaporation for the sake of efficient radiative heat transfer.

In the MBE process, a GaN buffer layer is deposited for 2 h at *T*_Subs_ = 720 °C on the GaN template after surface preparation (heating, degreasing). The GaN is grown under metal-rich conditions with beam equivalent pressure (BEP)_Ga_ = 4.5 × 10^–7^ mbar resulting in a growth rate of 6 nm/min. Any excess Ga is fully consumed before AlScN growth. The AlScN layers are grown with (BEP)_Sc_ = 1 × 10^–8^ ... 3 × 10^–8^ mbar and (BEP)_Al_ = 5 × 10^–8^ ... 8 × 10^–8^ mbar. Nitrogen-rich conditions with a metal-to-nitrogen ratio of ∼0.8 yield phase-pure wurtzite AlScN.^5^ Several 120–150 nm thick AlScN films were grown to reveal the effect of Sc composition on the structural properties of AlScN films. The Sc fraction is varied while ensuring a constant metal (Sc + Al) flux resulting in Sc contents in the range of 8–20% (cf. Figure 1e). Subsequently, an InGaN layer with a thickness of ∼100 nm is grown under metal-rich conditions at *T*_Subs_ = 600 °C on AlScN with a (BEP)_In_ of 3 × 10^–7^ mbar to prove the viability of the AlScN virtual substrate concept (referred to as sample 1 (S1)). A reference InGaN film is deposited directly on the regrown GaN using otherwise identical beam fluxes and substrate temperatures (referred to as sample 2 (S2)).

**Figure 1:**
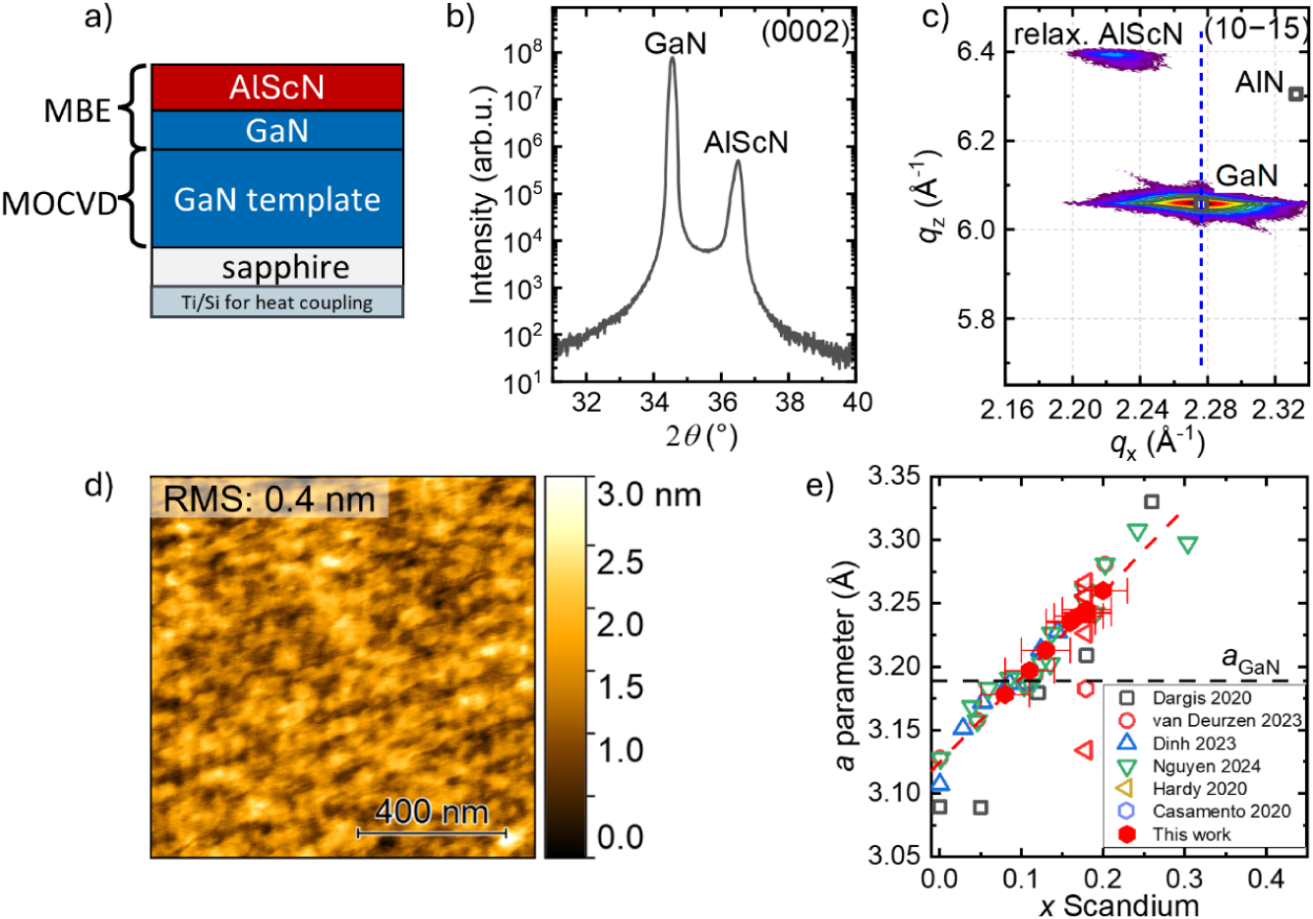
a) Schematic of the grown Al_0.8_Sc_0.2_N/GaN heterostructure. b) XRD 2θ-ω scan of the (0002) reflection of Al_0.8_Sc_0.2_N grown on GaN templates. c) A typical RSM of the (10–15) reflection of the Al_0.8_Sc_0.2_N/GaN heterostructure. The dashed blue line represents the in-plane lattice parameter of the GaN revealing a relaxed growth of AlScN on the GaN template. The black squares indicate the position of fully relaxed AlN. d) AFM scan of an Al_0.8_Sc_0.2_N film grown at *T*_Subs_ = 720 °C indicating an RMS roughness of 0.4 nm for a 1 × 1 μm^2^ area. e) Lattice parameters of Al_1–*x*_Sc_*x*_N samples grown at 720 °C vs Sc composition obtained from EDX scans (red hexagons) compared to literature values of MBE-grown AlScN (open symbols).^16,18–20,30,31^ The black dashed line represents the lattice constant of GaN, and the red dashed line is a guide to the eye.

High-resolution X-ray diffraction (HRXRD) on a Rigaku SmartLab diffractometer operating with a 9 kW rotating Cu anode provides initial insights into the structural properties of the heterostructures. The layer quality is determined by rocking curves of the symmetric (0002) reflection. Strain relaxation, lattice parameters, and composition of the AlScN and InGaN layers are extracted from reciprocal space map (RSM) data of the (10–15) reflection. The surface morphology is assessed by a Bruker Multimode 8 atomic force microscope (AFM) operating in the ScanAsyst Air mode using Bruker SCANASYST-AIR probes. Gwyddion software is used for image processing and deriving the root-mean-square (RMS) roughness.

Surface analyses using energy dispersive X-ray spectroscopy (EDX) were performed using a Zeiss MERLIN SEM operating under high vacuum conditions and equipped with an X-Max Extreme EDX detector running Oxford Instruments AZtec microanalysis software version 4.2. Unless otherwise specified, analyses were undertaken with a voltage of 10 kV and beam current of 450 pA.

For scanning transmission electron microscopy (STEM) investigations, electron-transparent cross-section lamellae were prepared using a JEOL JIB-4601F MultiBeam SEM-FIB system. STEM imaging and energy-dispersive X-ray spectroscopy (EDX) measurements were carried out using a double-aberration-corrected JEOL JEM-2200FS microscope operating at an accelerating voltage of 200 kV. Low-angle annular dark-field (LAADF) imaging was employed to achieve defect-sensitive contrast.

For the EDX measurements in TEM, an XFlash 5060 detector in combination with Bruker Esprit 2.3 software was used. Quantification was based on the Cliff–Lorimer method. Line scans were performed to determine the elemental concentrations of the different layers within the lamellae.

The photoluminescence (PL) is measured using a custom setup. A continuous wave (cw) HeCd laser (325 nm, 0.18 mW optical output power) is used for excitation. The PL signal is imaged onto the entrance slit of a 25 cm Czerny–Turner spectrograph by an achromatic lens where the spectrum is detected by a thermoelectrically cooled open-electrode Si charge-coupled device camera. All spectra are corrected for any background and the spectral response of the experimental setup.

## Results and Discussion

### MBE-Grown AlScN with Varied Sc Content

The structural properties of the layers are analyzed by XRD in Figure 1 and summarized with the AFM surface morphology. Figure 1a shows a sketch of the AlScN/GaN heterostructure. The XRD 2θ-ω scan of the (0002) reflection and a typical RSM of the (10–15) reflection of an Al_0.8_Sc_0.2_N film grown at *T*_Subs_ = 720 °C is shown in Figure 1b,c. The layer thickness is about 150 nm measured by X-ray reflectivity (XRR) and TEM. In the XRD 2θ–ω scan, the left and right peaks are from the underlying GaN template and the MBE-grown AlScN top layer. No other phases of AlScN are observed. The (0002) rocking curve (RC) full widths at half-maximum (fwhm) are 360 arcsec for the GaN and 400 arcsec for the Al_0.8_Sc_0.2_N layer (see Figure S1) reflecting the quality of the GaN template in terms of the tilt component.

We measured RSMs around the GaN (10–15) reflection to determine the lattice parameters of the Al_1–*x*_Sc_*x*_N layers. Figure 1c shows an exemplary RSM of a 150 nm-thick Al_0.8_Sc_0.2_N/GaN heterostructure. (A RSM around the GaN (10–15) reflection of a strained Al_0.89_Sc_0.11_N layer is shown in Figure S2. A single reflection related to a relaxed Al_0.8_Sc_0.2_N layer is observed in addition to the GaN peak. The in-plane and out-of-plane lattice parameters are derived from the *q*_*x*_ and *q*_*z*_ values of the RSM using the following equations:

1 $$q_{x}=2\pi \sqrt{\frac{4\left(h^{2}+hk+k^{2}\right)}{3a^{2}}}$$

2 $$q_{z}=2\pi \frac{l}{c}$$

resulting in *a* = 3.26 Å and *c* = 4.91 Å for relaxed AlScN film. These findings align well to values reported in ref 31. Figure 1d shows the corresponding AFM image of the AlScN surface. A low root-mean-square (RMS) roughness of 0.4 nm is found on 1 × 1 μm^2^, a value comparable to data reported in the literature.^16,31^

We put our values of the *a* lattice parameter in context to the literature data to visualize the feasibility of our virtual-substrate concept which mandates controlling the in-plane lattice parameter. Figure 1e shows the in-plane lattice constant *a* as a function of the Sc composition for Al_1–*x*_Sc_*x*_N samples grown at 720 °C (red hexagons). Sc compositions were determined by EDX for different Sc concentrations and linear interpolated. The red dotted line is a guide to the eye. The data are compared to data for MBE-grown AlScN (open symbols).^16,18–20,30,31^ As an overall trend, the reported *a* lattice constants monotonically increase with increasing Sc composition for all MBE-grown samples. Notably, all AlScN samples presented in this study are grown with a III/V ratio of about 0.8 at 720 °C.

### InGaN on AlScN Interlayers for High Indium Contents

The structural properties of two InGaN layers demonstrate the influence of the AlScN virtual substrate. Sample S1 features InGaN grown on AlScN (Figure 2a–d). This sample is compared to sample S2 where InGaN is grown directly on GaN (Figure 2e–h). We use identical growth conditions for both InGaN layers. Figure 2b displays the XRD ω-scans of the (0002) reflection of the GaN, the AlScN, and the InGaN layers. The fwhm values for sample S1 are 468 and 576 arcsec for the AlScN and the InGaN, respectively. The fwhm of the AlScN layer of sample S1 is slightly larger compared to the sample shown in Figure S1 due to a lower thickness of 120 nm. The fwhm value of the skew-symmetric (10–12)-reflection is 3400 arcsec for Al_0.8_Sc_0.2_N (cf. Figure S3). Dark field TEM images of the InGaN/AlScN and InGaN/GaN heterostructure is presented in the Supporting Information (cf. Figure S4). The In-content of *x*_In_ = 0.28 is extracted from the corresponding RSM (Figure 2c) and is consistent with values extracted from TEM-EDX (cf. Figure 3) and photoluminescence (cf. Figure 4). The fwhm of the XRD ω-scans of the (0002) for the InGaN layer of sample S2 in Figure 2f is 1370 arcsec and, therefore, significantly larger compared to the sample S1. The fwhm of the GaN template is approximately 360 arcsec in both cases. RSMs around the (10–15) asymmetric reflection providing information about the strain state are shown in Figure 2c,g for samples S1 and S2, respectively. The tilted dashed lines indicate the fully relaxed state (in this case InGaN). The other vertical dashed line in Figure 2c passing through the AlScN reflection indicates the strained state for InGaN grown on the AlScN virtual substrate.

**Figure 2:**
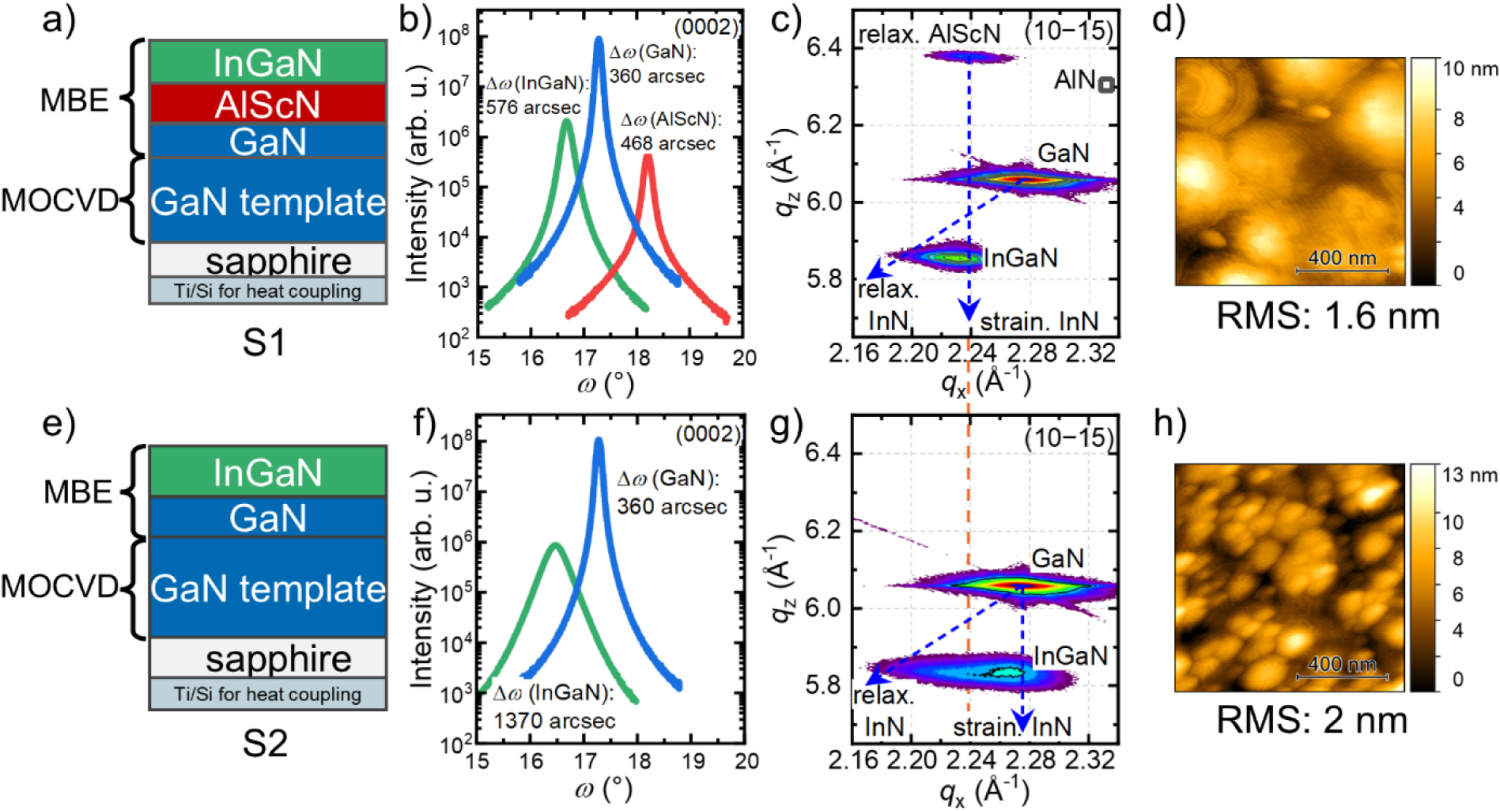
Sample structures of sample a) S1 and e) S2. b,f) XRD ω-scans of the (0002) reflection. The full width at half-maximum for the InGaN grown on AlScN is 576 arcsec and 1370 arcsec for the InGaN directly grown on GaN. c,g) Reciprocal space maps around the (10–15) reflection of In_0.28_Ga_0.72_N grown on the AlScN pseudosubstrate. Dashed straight and diagonal lines correspond to the position of the InGaN diffraction points assuming that InGaN is strained or relaxed on AlScN or GaN, respectively. In Figure 2c, the RSM reveals a nearly strained In_0.28_Ga_0.72_N on AlScN. g) Reciprocal space map of In_0.25_Ga_0.75_N directly grown on GaN (w/o AlScN) showing increasing relaxation of the lattice, accompanied by structural deterioration (see Figure 2b,f), pointing out the improved structural quality of InGaN when using an AlScN pseudosubstrate. d,h) AFM scans of d) In_0.28_Ga_0.72_N on AlScN and h) In_0.25_Ga_0.75_N on GaN.

**Figure 3:**
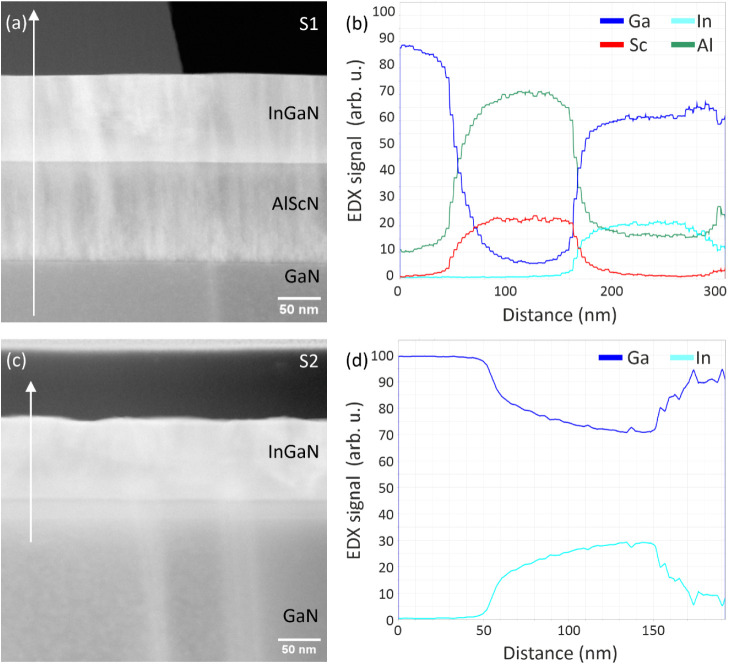
a) TEM image of InGaN/AlScN (sample S1) and c) InGaN/GaN (sample S2). The defect-sensitive LAADF images show a higher number of defects at the AlScN/GaN interface, most likely due to strain relaxation. The EDX line scans of sample S1 depicted in (b) show a homogeneous distribution of Sc (red curve) and In (cyan curve). The Sc composition is about *x*_Sc_ = 0.25 ± 0.1, and the In composition is *x*_In_ = 0.25 ± 0.05. The corresponding LAADF image of S2 is shown in (c). In contrast to S1, the EDX line scans of sample S2 depicted in (d) show a strong gradient in the growth direction due to the compositional pulling effect.

**Figure 4:**
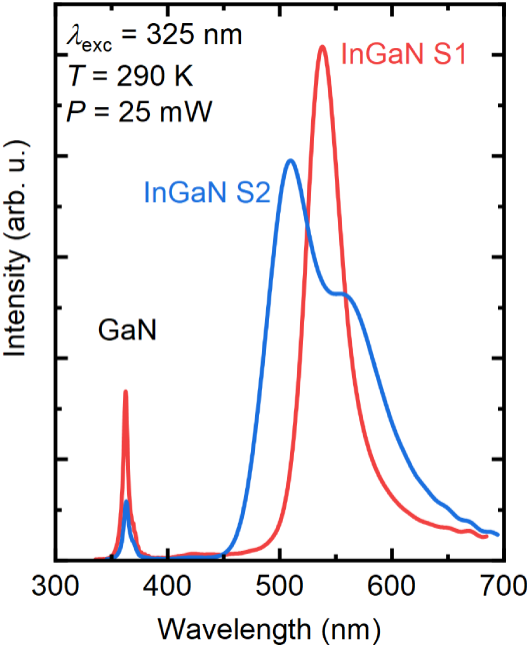
Room temperature photoluminescence spectra of InGaN/AlScN and InGaN/GaN structures (samples S1 and S2).

The average In mole fraction of the layers and their strain state are extracted from the *a* and *c* lattice parameters (eqs 1 and 2) obtained from the RSMs assuming biaxial strain.^32^ The lattice parameters *a*_GaN_ = 3.189 Å, *c*_GaN_ = 5.185 Å for GaN, *a*_InN_ = 3.545 Å, *c*_InN_ = 5.703 Å for InN, and the elastic constants for GaN and InN were used according to ref 33. The red dotted line between Figure 2c,g shows a direct comparison between the fully strained states using AlScN or GaN as a substrate.

This direct comparison qualitatively evidence that the larger in-plane lattice constant of the AlScN shifted the associated reflection to lower reciprocal lattice units. This results in a reduced lattice mismatch of the InGaN layer to the AlScN (pseudo) substrate in sample S1 compared to the lattice mismatch of InGaN to GaN in sample S2. Consequently, the reduced screw-/mixed-type dislocation density in the former sample is strongly reflected by the value of the fwhm of its (0002) rocking curve (cf. Figure 2b) which is much lower than those for InGaN grown on GaN, both in our work as well as reported in the literature, e.g., in ref 34.

The RSM of the InGaN grown directly on GaN (sample S2) is shown in Figure 2g and gives a *x*_In_ = 0.25 (point of highest intensity). The RSM of sample S2 shows an increasing relaxation of the lattice accompanied by a strong gradient of the In composition. This is indicated by the significant broadening of the InGaN reflection to lower reciprocal lattice units. We attribute this to the compositional pulling effect: the deformation energy caused by the stress drives the indium atoms incorporated in the crystalline layer to the surface of InGaN epilayers. Consequently, the indium composition of InGaN epilayers increases with proceeding relaxation along the growth direction, the *c*-axis.^35^ This process and an increase of the mosaicity of the layers is reflected by the broad fwhm of the ω-scan of the (0002) reflection of 1360 arcsec.

The lower lattice mismatch of the InGaN layer to the AlScN virtual substrate completely eliminates any hints of compositional pulling in sample S1, highlighting the improved structural quality of InGaN when using an AlScN pseudosubstrate.

We also calculate the in-plane strain $\varepsilon_{∥}=\frac{a-a_{r}}{a_{r}}$ where *a* is the lattice constant of the InGaN layer derived from the RSMs and *a*_*r*_ the lattice constant of fully relaxed InGaN with this composition (according to Vegard’s law). The in-plane lattice parameter of *a =* 3.25 Å of the InGaN in sample S1 exhibits −0.01 of compressive strain. The in-plane strain (calculated for the point of highest intensity) for sample S2 reaches a value of −0.02, which is double that of sample S1. In other words, the strain relaxation—accompanied by a lower dislocation density—is less severe in sample S1 due to the lower lattice mismatch between InGaN and AlScN.

To put the quality of our InGaN material into context, we apply the established analysis for growth on GaN without the AlScN pseudosubstrate. Evaluating the RSM for sample S1 yields a relaxation degree of 20%, considerably lower than values reported for epitaxial InGaN grown on GaN templates.^34,36^ However, this value strongly depends on the lattice parameter of the substrate and no published values for the relaxation state of InGaN on AlScN are available to date. The RSM of the InGaN grown directly on GaN (sample S2) is shown in Figure 2g. It features a *x*_In_ = 0.25 (point of highest intensity) and seemingly implies a similar relaxation degree of 20%. This similar value may be misleading but is not at all comparable to the relaxation state of sample S1 as both samples have very different substrates with very different in-plane lattice constants. Consequently, the absolute strain relaxation in terms of change in *a* lattice parameter and the accompanied higher dislocation density in sample S2 are due to its larger lattice mismatch, illustrating that the use of AlScN pseudosubstrates is highly favorable. To illustrate the relevance of AlScN pseudosubstrates, we present XRD RSM results of InGaN/AlScN and InGaN/GaN heterostructures for different Sc and In composition in Figure S5.

The surface morphology obtained by AFM for samples S1 and S2 are displayed in Figure 2d,h, respectively. The surface of S1 shows typical hexagonal pyramids and an RMS roughness of 1.6 nm on a 1 × 1 μm^2^ area. The surface of sample S2 exhibits a higher density of hillocks, likely caused by a higher density of dislocations with screw-component as is evident from the broader (0002) rocking curve.

The structural quality of the layers is analyzed by STEM imaging. Figure 3a shows a defect-sensitive LAADF-STEM image of the InGaN/AlScN/GaN heterostructure with *x*_In_ = 0.28. The number of defects is high at the AlScN/GaN interface due to strain relaxation and is decreasing toward the InGaN/AlScN interface. The qualitative EDX line profile across section of the InGaN/AlScN sample confirms the homogeneous In content (cyan curve) of the InGaN layer (Figure 3b). Since transmitted electrons can reach the Al_2_O_3_ substrate and generate characteristic X-rays, the Al profile (green curve) erroneously shows the presence of Al even in the Ga containing layers. A quantitative STEM-EDX analysis, removing the spurious Al, yields an indium content of *x*_In_ = 0.25 ± 0.05, which is in excellent agreement with the value obtained from HRXRD (Figure 2c). However, this inaccuracy in the Al content in turn leads to a rather inaccurate determination of the Sc content to 0.25 ± 0.10.

The corresponding defect sensitive LAADF image of sample S2 is depicted in Figure 3c. In contrast to S1, the EDX analysis of sample S2 shows a strong gradient of the In composition along the growth direction (Figure 3d)). The In composition is increasing from about *x*_In_ = 0.2 to *x*_In_ = 0.3. This is assigned to the compositional pulling effect as also observed in the RSM of this sample (Figure 2g).

Room-temperature PL data for both InGaN samples are presented in Figure 4. The emission spectrum of sample S1 shows a distinct peak at 538 nm (2.32 eV) with a full width at half-maximum of 29 nm (121 meV) that can be adequately fitted with an asymmetric double sigmoid function. The asymmetric broadening of the emission peak is indicative of alloy disorder leading to carrier localization in a ternary compound such as InGaN.^37^ The observation of a single emission line confirms the EDX measurements of a homogeneous indium incorporation along the growth direction. As there are no PL data for InGaN on AlScN available, we compare our results to InGaN grown on GaN and other substrates than GaN for the same growth technique. For example, the fwhm of the InGaN luminescence peak of sample S1 is comparable or even narrower than films grown on GaN^34,38^ or spinel.^39^

Sample S2 shows a completely different emission pattern featuring two clearly visible separate peaks (blue curve). The maxima are found at 506 and 565 nm (2.45 and 2.19 eV) featuring fwhm’s of 34 and 83 nm (165 and 340 meV), respectively. The high-energy peak results from the compressively strained InGaN close to the GaN template interface, while the low-energy peak likely stems from the relaxed region that incorporates a higher content of indium due to the compositional pulling effect. The origin of this double peak has been discussed as early as 2002 by Pereira et al.^40^ and has been reported even for samples exhibiting a gradual change in indium incorporation as evidenced by XRD reciprocal space maps.^41^ The occurrence of a gradual increase of indium content during the progressive relaxation of the InGaN film^36^ is in good agreement with a distinct double peak in the luminescence spectrum. A conservative estimate of the diffusion length using a carrier lifetime of 100 ps and an ambipolar diffusion coefficient of 1 cm^2^/s^42^ yields a value of 100 nm, which is on the same order of magnitude as the layer thickness. Consequently, it is plausible that carriers generated in the graded indium layer may diffuse and/or drift into the high-indium content region at the surface, where they recombine at lowest potential energy.

## Conclusion

In summary, we have demonstrated the potential of using Al_1–*x*_Sc_*x*_N as a pseudosubstrate to grow InGaN with high In concentration (*x*_In_ = 0.28). We modify the in-plane lattice parameter of 120 nm thick, phase pure Al_1–*x*_Sc_*x*_N layers with 0.08 < *x*_Sc_ < 0.2 for subsequent high indium content In_0.28_Ga_0.72_N layers. An improved lattice matching due to AlScN pseudosubstrates leads to an improved structural quality of InGaN. These layers show a higher structural quality as indicated by narrower XRD rocking curves and a narrow PL emission around 538 nm when using Al_1–*x*_Sc_*x*_N as a pseudosubstrate, while compositional pulling is eliminated. Further tuning the Al_1–*x*_Sc_*x*_N lattice constant to even larger values would enable virtual substrates for the growth of lattice-matched InGaN tailored for red-emitting InGaN micro-LEDs or even beyond, exploiting the vast potential of high-In-content InGaN alloys whose band gap energies may approach the telecom wavelength windows.

## Supplementary Material



## Notes

Structural characterization by XRD and TEM; RSMs (PDF)
