# Supplementary material for: AlScN Pseudosubstrates for High Indium Content InGaN Alloy Epitaxy
Source: ACS Appl Mater Interfaces. 2025 Oct 23;17(44):61445–52. doi: 10.1021/acsami.5c14209 (PMC12598697; doi:10.1021/acsami.5c14209)
Supplement: Supplementary file 1 [file am5c14209_si_001.pdf]

Supporting Information

AlScN pseudo-substrates for high indium content InGaN alloy epitaxy

Jörg Schörmann,[1,*] Mario F. Zscherp,[1] Silas A. Jentsch,[1] Martin Becker,[1] Markus Stein,[1] Florian Meierhofer,[2] Christoph Margenfeld,[2] Fabian Winkler,[3] Andreas Beyer,[3] Kerstin Volz,[3] Andreas Waag,[2] and Sangam Chatterjee[1]

[1]Institute of Experimental Physics I and Center for Materials Research, Justus-Liebig-University Giessen, D-35392 Giessen, Germany
[2]Nitride Technology Center, Institute of Semiconductor Technology, Technische Universität Braunschweig, D-38106 Braunschweig, Germany
[3]Materials Science Center and Faculty of Physics, Philipps-University Marburg, D-35032 Marburg, Germany

*joerg.schoermann@exp1.physik.uni-giessen.de

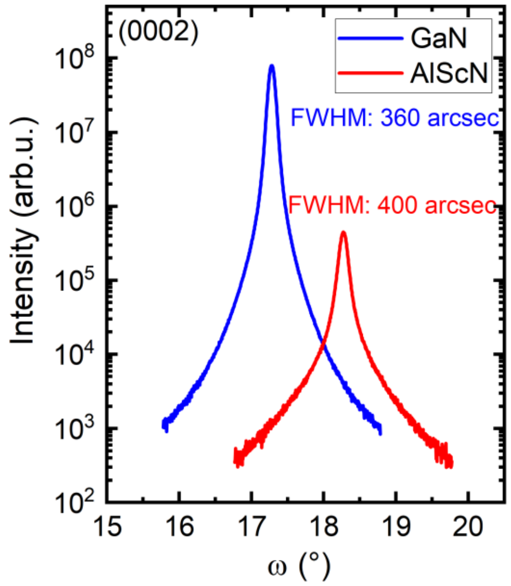

Figure S1. XRD ω-scans of the (0002) reflection. The full width at half maximum for the GaN is 360 arcsec and 400 arcsec for the $Al_{0.8}Sc_{0.2}N$.

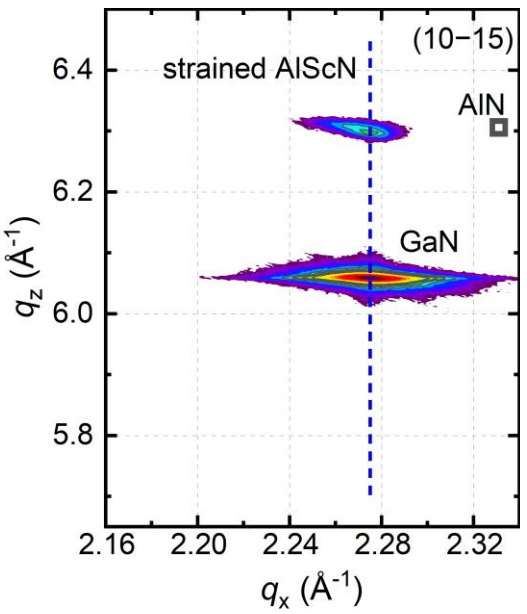

Figure S2. A typical RSM of the (10−15) reflection of the $Al_{0.89}Sc_{0.11}N$ /GaN heterostructure. The dashed blue line represents the in-plane lattice parameter of the GaN revealing a strained growth of AlScN on the GaN template. The black square squares indicate the position of fully relaxed AlN.

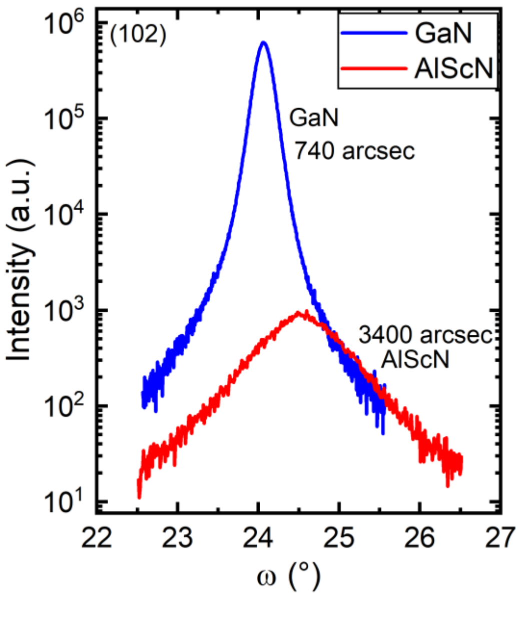

Figure S3. XRD ω-scans of the (10−12)-reflection. The full width at half maximum for the GaN is 740 arcsec and 3400 arcsec for the $Al_{0.8}Sc_{0.2}N$.

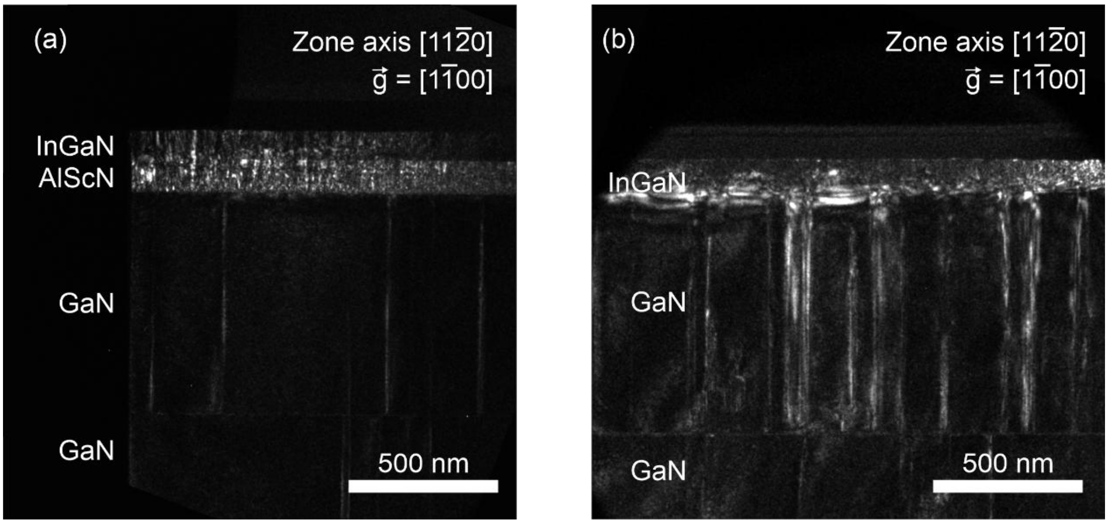

Figure S4. Dark field TEM images of a) InGaN/AlScN/GaN and b) InGaN/GaN heterostructures. In comparison to the GaN layers, the AlScN film in a) and the InGaN film in b) is substantially more defective resulting in complex contrast in the TEM image. This contrast is associated with a large density of structural defects emerging close to the AlScN/GaN or InGaN/GaN interface. Based on the comparably narrow (0002) rocking curve and the strong broadened (10-12) reflection, those defects are expected to be largely edge-type dislocations. In b) the InGaN shows a bright contrast at the InGaN/GaN interface, most likely due to strong compressive strain.

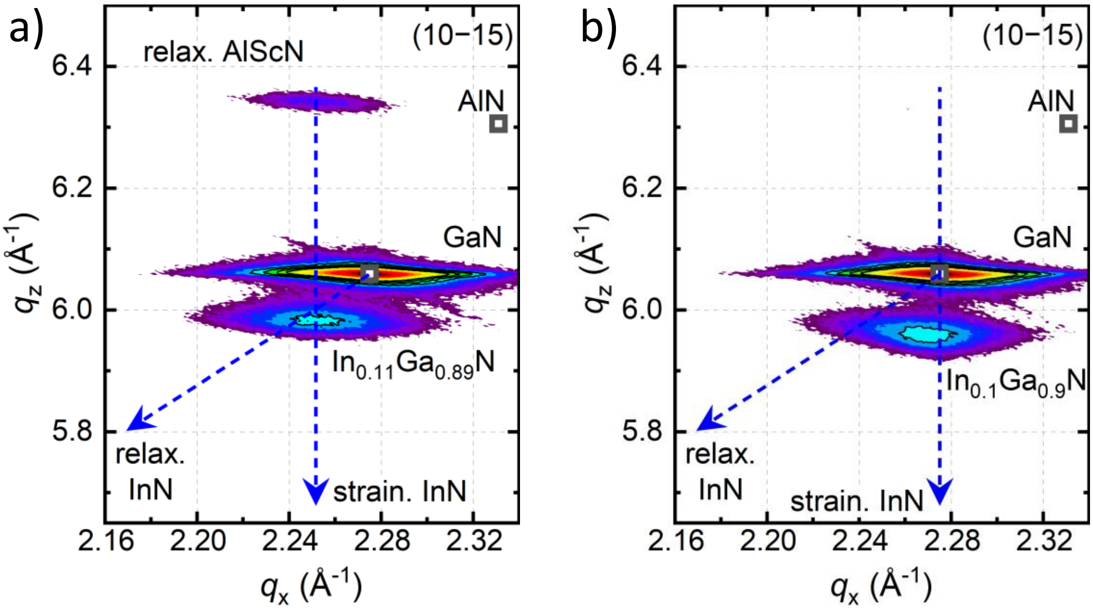

Figure S5. a) RSM of the (10−15) reflection of InGaN on $Al_{0.87}Sc_{0.13}N$. The dashed blue line represents the in-plane lattice parameter of the AlScN revealing a pseudomorphic growth of $In_{0.11}Ga_{0.89}N$ on the AlScN pseudo-substrate. The black square squares indicate the position of fully relaxed AlN. b) RSM of the (10−15) reflection of $In_{0.1}Ga_{0.9}N$ on GaN. The dashed blue line represents the in-plane lattice parameter of the GaN revealing a partially relaxed growth of InGaN on the GaN substrate.
